# Whole-Cell Thermal Sensor for the Detection of *P. falciparum*-Infected Erythrocytes: Imprinted Polymers as Synthetic Receptors for the Detection of Malaria

**DOI:** 10.1021/acssensors.4c02706

**Published:** 2025-01-16

**Authors:** Rocio Arreguin-Campos, Ramayana M. Medeiros Brito, Ana Rafaela Antunes Porto, Augusto César Parreiras de Jesus, Lilian Lacerda Bueno, Ricardo Toshio Fujiwara, Hanne Diliën, Thomas J. Cleij, Kasper Eersels, Bart van Grinsven

**Affiliations:** † Sensor Engineering Department, Faculty of Science and Engineering, Maastricht University, P.O. Box 616, 6200 MDMaastricht, The Netherlands; ‡ Department of Parasitology, Institute of Biological Sciences, Federal University of Minas Gerais, 31270-901Belo Horizonte, Brazil; § Post-Graduate Program in Health Sciences: Infectious Diseases and Tropical Medicine, Faculty of Medicine, Federal University of Minas Gerais, 30130-100Belo Horizonte, Brazil

**Keywords:** biomimetic sensing, malaria, infectious disease, point of care, imprinted polymers, graphene oxide

## Abstract

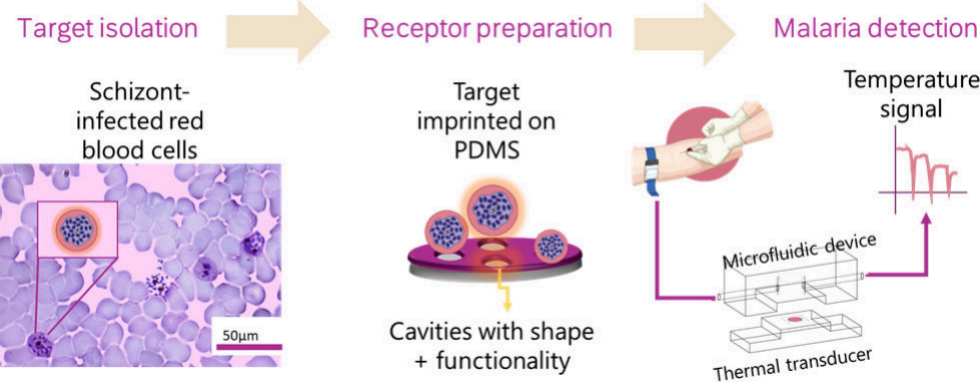

Malaria is a major public healthcare concern worldwide, representing a leading cause of death in specific regions. The gold standard for diagnosis is microscopic analysis, but this requires a laboratory setting, trained staff, and infrastructure and is therefore typically slow and dependent on the experience of the technician. This study introduces, for the first time, a biomimetic sensing platform for the direct detection of the disease. The heart of the sensor consists of a synthetic receptor created by surface imprinting *Plasmodium falciparum*-infected erythrocytes in a polymeric matrix. This receptor layer is coupled to a low-cost and fast thermal transducer platform, allowing for real time detection of the infection. The artificial receptor is a composite of poly(dimethylsiloxane) and graphene oxide that, in combination with the thermal readout, exhibited a limit of detection below 0.5% parasitemia in erythrocyte samples. This value is relevant for the diagnosis of infection in patients with low parasitemia levels. This study proves that biomimetic sensors could be further explored for the development of point-of-care technologies for the management of the disease.

Malaria is a life-threatening illness that remains one of the most impactful infectious diseases worldwide. It originates from five *Plasmodium* blood parasite species: *P. malariae*, *P. ovale* and *P. knowlesi, P. falciparum* and *P. vivax*, with the two latter ones being the main causative agents in Africa and South America. According to the Malaria report of the World Health Organization, the annual incidence of cases is around 250 million infections and 600 000 deaths globally, with 94% of all cases and deaths in the African region.^1^

Accurate diagnosis of the infection can prevent further transmission and deaths related to the disease. The field standard technique is light microscopy through blood smears, which is highly sensitive. Nonetheless, the accuracy of the test relies completely on the competence of the technician, and specific high costs of equipment and training are essential.^2^ Besides the traditional examination, other diagnostic tools such as polymerase chain reaction (PCR), and serological tests (e.g., ELISA tests) provide enhanced accuracy and reliability for detecting mixed infections. However, they are not available for large-scale application and they are in general not suitable for routine monitoring linked to the high cost for implementing these techniques.^3^ Rapid diagnostic tests (RDTs) that rely on the detection of biomarkers that are present in high concentrations in the blood of infected patients are commercially available in diverse formats.^4,5^ Some benefits related to these tools are the speed of results, their suitability to be used in remote areas, and the fact that they do not require highly skilled personnel for performing them. However, they are based on biological receptors, which limits their performance to specific conditions that are adequate for them (pH, temperature). Therefore, point-of-care (PoC) technologies for malaria detection face the challenging task of combining reliability in their performance by targeting direct identification of the infection while at the same time remaining simple and cost-effective. Devices that have these attributes could be fundamental in the management of the disease.

Biosensors that rely on synthetic receptors have attracted attention due to the performance they are able of maintaining in different physicochemical conditions and in complex samples.^6^ Imprinted polymers (IPs), for instance, are polymeric networks that mimic natural receptors. Their recognition ability is based on nano- and microcavities created within the material that match the desired target in terms of geometry and complementarity of chemical functionalities, resembling the lock and key mechanism present in nature (*e.g*. enzymes and antibodies).^7^ By coupling them with the adequate transducing platform they possess the potential of meeting the requirements or diverse application fields.^8^ Although artificial receptors are commercially available for a wide range of molecular targets,^9,10^ the development for targets such as bacteria, cells, or parasites remains in research stage and still hampers the potential that the materials hold for their use in disease diagnosis.

Some of the challenges that IPs face when being tailored for big analytes are the need to create the active cavities on the surface of the materials to facilitate the mobility of the analyte and therefore the binding event. Experimental protocols are therefore complex and multistep. Moreover, imprinting conditions should be optimized in order to maintain the features of the target as much as possible.^11^ These are some reasons why polymer imprinting has been limited to a selection of larger targets including proteins, bacteria, and a few types of cells^12,13^ (Supporting Information (SI), Table S1). The development of synthetic receptors for parasites, such as the *Plasmodium* spp. genus, could have a sizable impact regarding the diagnosis of the malaria disease. So far, no approach has been investigated for preparing such materials, and therefore, no biosensor targeting the whole-infected blood cells has been reported.

In the present study, we explore the use of surface imprinted polydimethylsiloxane (PDMS) as synthetic receptors for *P. falciparum*. The unique strategy takes into consideration that the parasite in its diagnostic stage is mostly present within infected red blood cells. In terms of materials, the use of PDMS ensures the chemical and mechanical robustness that makes it so popular in lithographic applications.^14,15^ Furthermore, the receptor is doped with graphene oxide (PDMS-GO) as a thermal additive in order to enhance the thermal conductivity of the layer, facilitating thermal transduction.^16^ The heat transfer method (HTM) is based on the registration of changes in interfacial thermal resistance that result from receptor–analyte binding events. The sensor requires little instrumentation in comparison with the widespread readout technologies such as electrochemical platforms and functions as a low-cost temperature control unit and a transducer at the same time.^17,18^ In recent research, the combination of imprinted polymers with HTM has proven to result in sensors that are promising tools for PoC monitoring.^19,20^ The user-friendly nature of the sensor, its rapid response time, and the straightforward and low-cost synthesis process of highly resistant synthetic receptors are advantages that could make it a technology suitable for resource-limited settings, a key element in malaria diagnosis. The study presented here reports, to our knowledge, the first direct biomimetic sensor for malaria, allowing the real-time detection of the parasite without the need for labels at a relevant limit of detection for the diagnosis of the infection.

## Materials and Methods

A detailed description of the methods is provided in SI, section S2.

### Blood Sample Preparation and Parasite *in Vitro* Culture

*P. falciparum* parasites previously frozen were thawed, transferred to culture flasks, and supplemented with 10% of heat inactivated human serum type O+. Noninfected O+ RBCs were added to complete the final hematocrit to 10%. The flasks containing the parasites were kept under microaerophilic conditions at 37 °C. Parasitemia was monitored daily by blood smears stained by the Giemsa dye method and examined under an optical microscope to determine the percentage of parasitized RBCs (SI, Figure S1), using the ratio between the number of infected and noninfected erythrocytes. To separate the *P. falciparum* mature schizont stages, a four-step Percoll gradient was followed.^21^ The infected cells were used to prepare the surface imprinted polymers.

### Preparation of Synthetic Receptors

A resin containing polydimethylsiloxane base and graphene oxide powder was prepared (0.01%). The base-GO was mixed with the curing agent following the ratio (10:1 (w/w)), and the mixture was further diluted in tetrahydrofuran (10% w/w) aluminum squares (size of 1 cm^2^) were spin-coated for 60 s at 5000 rpm with 150 μL of the prepared PDMS-GO stock solutions. The thin layers were precured for 10 min at 65 °C. Afterward, the substrates were placed onto a flat surface at room temperature and the parasite suspension (39 500 cells in 200 μL of 1× PBS buffer) was applied as a droplet on the polymers and left to sediment for 20 min. The substrates with the droplet were then placed back in the oven at 65 °C for 4 h in order to finalize the curing of the PDMS. The films were washed with water first to rinse the residues of salts on the surface, followed by ammonium–chloride–potassium (ACK) buffer to lyse the red blood cells and make the imprinted cavities free. Optical characterization was performed with brightfield and scanning electron Microscopy.

### Heat Transfer Method Measurements for the Detection of Malaria

The biosensor setup has been described previously.^17^ Red blood cells (O+) containing *P. falciparum* schizonts were adjusted with fresh noninfected erythrocytes to obtain a parasitemia of 5%. These samples were further diluted with 1× PBS in order to obtain 10 mL of parasite solution at a concentration of 0.5% parasites per mL and hematocrit of 10%.

For each experiment, injections are carried out at a flow rate of 2 mL per minute for 2 min while continuously monitoring the temperature and thermal resistance for 20 min at the solid/liquid interface (of the receptor and the infected blood) in order to register the signal when the binding event takes place.

## Results and Discussion

### Optical Characterization of Imprints

**Figure 1:**
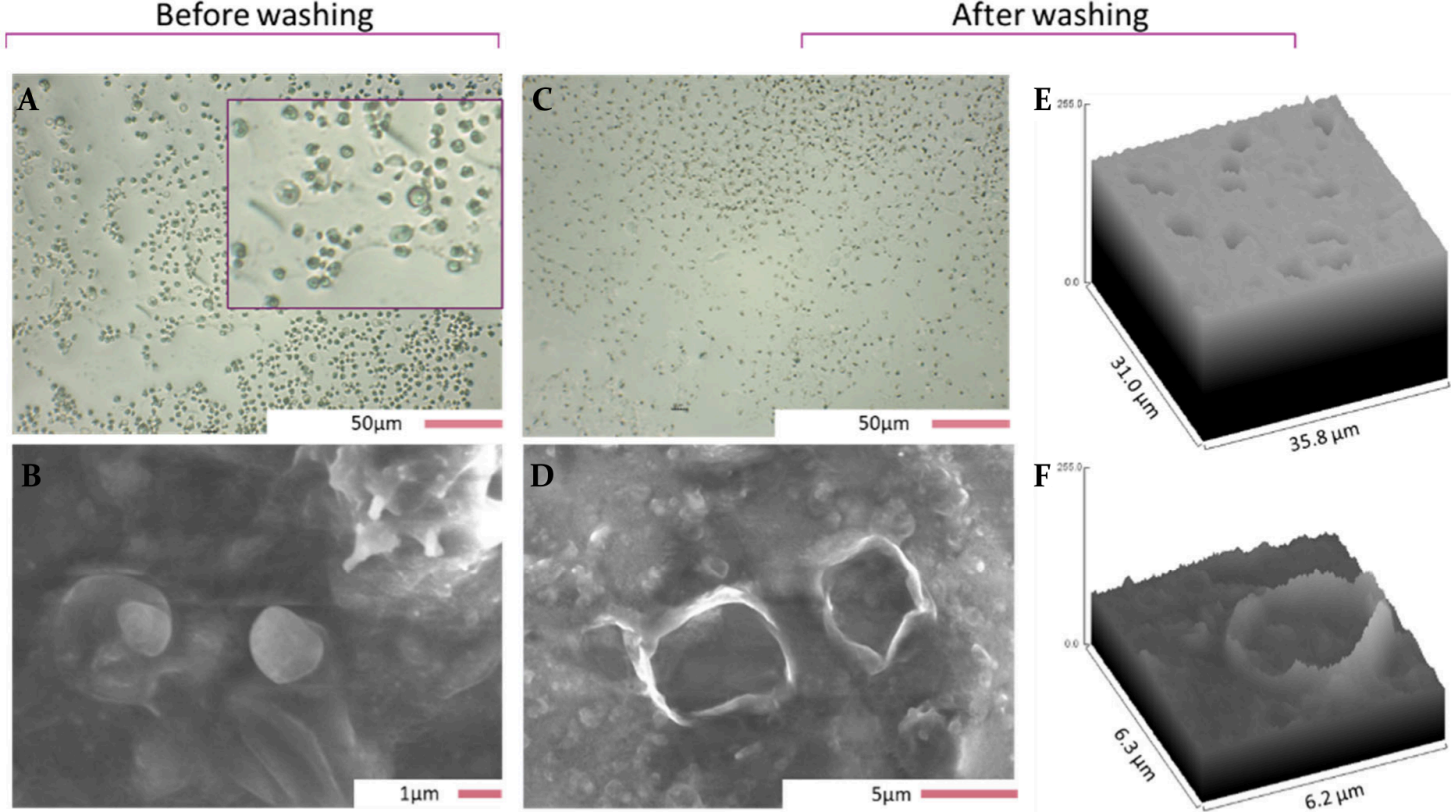
Optical characterization of polymeric imprints on glass. (A) Brightfield picture of unwashed imprinted PDMS exhibiting the distribution of schizont-infected cells on the layers. (B) SEM image from released merozoite on the PDMS receptors. (C) Brightfield image of washed layers. (D) SEM featuring empty microcavities on the materials. (E,F) surface plots of the polymeric materials after rinsing off the template displaying the imprints on the PDMS-GO (constructed from brightfield and scanning-electron microscopy).

A simple sedimentation-based surface imprinting technique was employed to prepare artificial receptors with parasitized RBCs. The strategy hereby employed to prepare RBC imprints offers several advantages over the most commonly used imprinting techniques (microcontact stamping,^22^ molding,^23^ and emulsion imprinting^24^), such as not requiring a stamp for the deposition of the analyte onto the polymer’s surface and therefore ensuring that all the template stays on the receptor in order to create the recognition sites. In order to confirm the presence of functional cavities on the materials, brightfield microscopy was employed first to ensure that the schizont-infected RBCs adhered to the PDMS-GO after the imprinting process. Figure 1A allows the visualization of the round iRBCs containing merozoites in a darker shade in a wide arrange of sizes (average 3.1 ± 1 μm), which aligns to the reported dimensions of parasitized erythrocytes.^25,26^ Furthermore, the distribution of the target across the prepared layers can be observed. While homogeneous in some areas, other spots are less covered, which is related to the imprinting technique employed, where the target freely assembles by gravity, leading to the obtained pattern. In previous research, similar results were obtained for bacteria imprinted PDMS-GO layers, demonstrating that this has little effect on the sensor performance as long as the average surface coverage is constant.^16^ Surface coverage indicates the proportion of area that is occupied by the template and was found to be 15.8 ± 3.1% (calculated with ImageJ), which aligns with the reported functionalized area for other microtargets such as bacteria.^16,27^ Moreover, we hypothesized that some schizonts would rupture during the imprinting process, leading to free parasite deposits on the layers. This was confirmed with scanning electron microscopy, shown in Figure 1B, where a released merozoite can be observed on the polymer. In order to ensure that the receptor will be able to rebind the target after the imprinting process, it is necessary to remove the target off the layer to make the cavities available. Figure 1C displays the change on the surface upon lysing and rinsing the iRBC from the PDMS films, exhibiting dark areas that correspond to the empty pockets in the material. Zooming in on the washed binding cavities with SEM, it can be established that they match in morphology and size to the schizont-infected RBCs employed for the imprinting (Figure 1D). An additional remark about SEM images is that they also allow the visualization of some lumps on the surface of the materials, which are derived from the imprinting process in which some salts can deposit and be covered by the PDMS-GO resin. Finally, parts E and F of Figure 1 depict the surface plots obtained by ImageJ that further allow the visualization of the empty microcraters on the polymeric receptors. When comparing these images, it can be highlighted once again that the dimensions of these empty cavities (∼4 μm) are comparable to the size of the iRBCs originally deposited on the layers. This finding supports that the integrity of the morphological characteristics of the target was maintained during imprinting and after washing, which is relevant for the hypothesis that the cavities will be able to rebind iRBCs. Additional pictures of the imprints are provided in SI, Figure S2.

### Infected Blood Thermal Measurements Employing Synthetic Receptors

**Figure 2:**
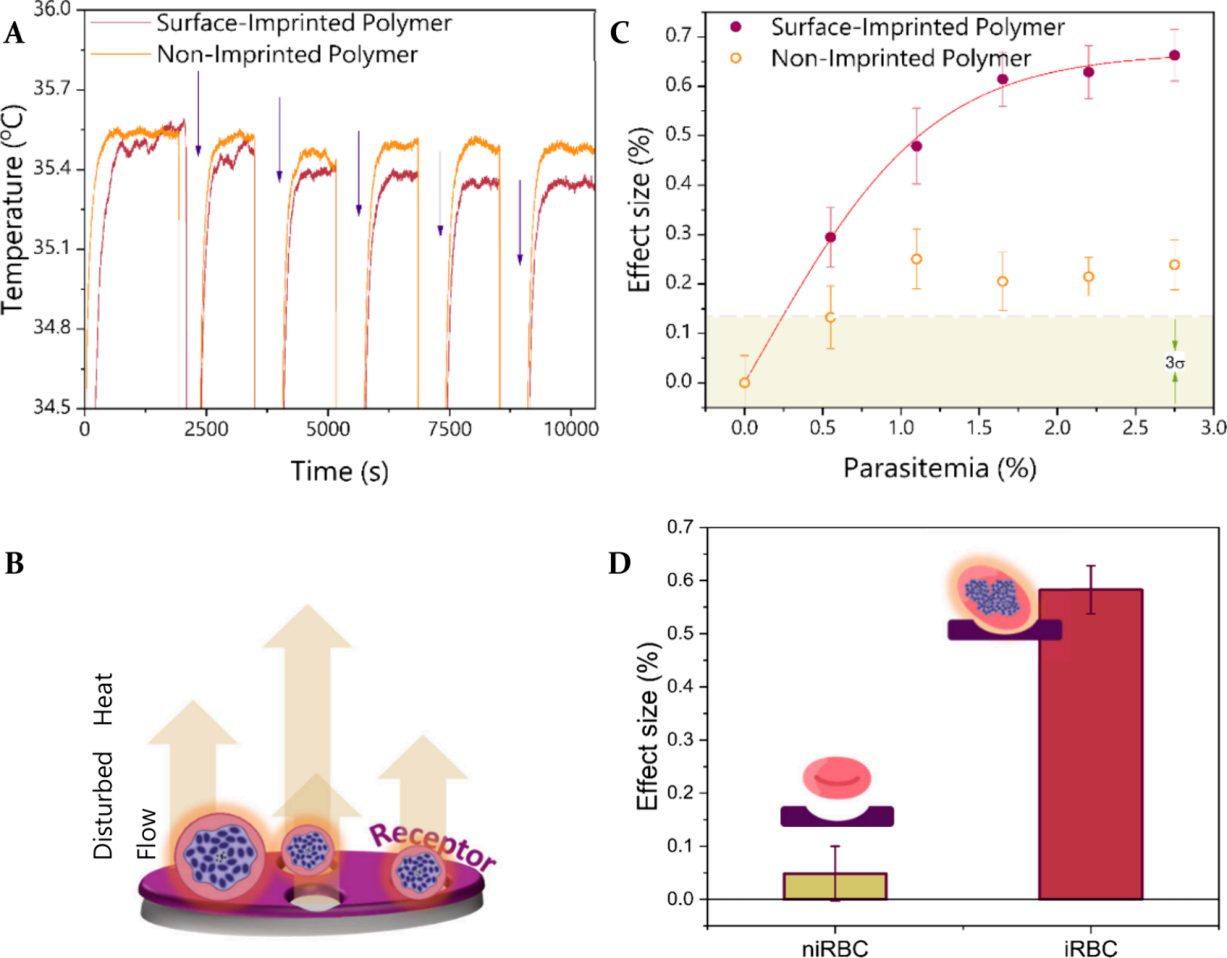
Quantitative thermal response of the sensor. (A) Representative temperature response of the synthetic receptors where each arrow indicates injection of the iRBCs (0.5%) parasitemia. (B) Schematic representation of the synthetic receptors upon the binding of the target. (C) Dose–response curves obtained from six independent replicate experiments where the noise bars reflect the signal variability and the light green area the noise of the equipment. (D) Comparison of the sensor’s response toward infected RBCs and healthy erythrocytes (niRBC), where error bars reflect variability from four independent replicates.


The obtained functionalized PDMS-GO layers were assessed for the quantification of *P. falciparum*-infected samples with the heat transfer method employing a microfluidic flow cell, which is described in the SI (Figure S3). A suspension of noninfected RBCs in buffer was employed to measure a baseline for the quantitative experiments by filling the microfluidic chamber and allowing the system to reach an equilibrium temperature. The liquid was then replaced with iRBCs in buffer at a concentration of 0.5% parasitemia and stabilized for 30 min. The injection process is repeated, and the temperature is monitored real-time employing a thermocouple that is placed above the synthetic receptor in the setup. Figure 2A shows the representative raw temperature signals observed when a chip covered with the synthetic receptor (surface-imprinted polymer-SIP) and a reference one covered with a control receptor (non-imprinted polymer-NIP) were added to a solution containing iRBCs. This experiment shows that the transmitted temperature drops over time in a more predominant way for the curve corresponding to the SIP, which can be directly related to the binding event of the iRBCs to the synthetic receptors derived from the geometrical and chemical complementarity of the cavities and the target. Upon capture, the target disturbs the heat flow at the interface of the PDMS layers by blocking the free cavities present on the material (Figure 2B). The temperature data for each addition are averaged upon stabilizing for each parasitemia concentration (*T*_c_) and an effect size can be calculated, reflecting the percentage of signal change that is obtained for every concentration when comparing to the baseline (T1).

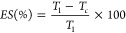


The average *ES* for each concentration was calculated for six independent samples and used to construct a dose–response curve. From these data, presented in Figure 2C, it is evident that the sensor is able to provide a concentration-dependent response when the synthetic receptors are exposed to the target. When contrasting to the control PDMS-GO layers, the results show that the SIPs provide an effect that is around three times higher at the end of the additions, confirming that the obtained results can be attributed to the presence of functional pockets on the artificial receptors. It is also of interest that the control layers provide a response above the noise mark of the sensor (in light green), indicating that the iRBCs possess affinity toward the material, leading to adhesion. This observation can be linked to the morphological modifications that are induced on the cells by the presence of the parasite. It has been suggested that the malaria inserts proteins into the membrane of the host cell, resulting into deformations called knobs, which are important for the adherence of the erythrocytes to the vascular endothelium.^28^ This is reflected on the sensor’s signal; however, the adhesion reaches a maximum and remains stable at a low ES, indicating that this nonspecific behavior can be filtered out by determining the correct cutoff response for future sensing applications. The curve obtained for the SIP layers can be fitted to a dose–response equation that describes the observed trend. The first injections (from 0% to 1.6% parasitemia) provide a linear signal increase derived from the progressive addition of target to the receptors, until reaching a plateau, which indicates that the cavities have been filled and now the layer is saturated, leading to a constant signal from the HTM. The limit of detection was calculated following the 3σ method taking into consideration the averaged noise obtained for the measurements, resulting in a theoretical LoD of 0.25% and an experimental LoD of 0.5% parasitemia. When employing microscopy for the examination of contaminated blood specimens, parasitemia in a patient is considered to be low when it is found in quantities <1%.^29^ On the other side, hyperparasitemia is defined for values >2% in low density transmission areas and >5% in areas of high stable malaria transmission intensity.^30^ Defining the level of infection serves as a guideline for accurately determining the treatment of the patient. These values would indicate that the sensor is able to detect the infection at low concentrations of infected RBCs and along the range toward hyperparasitemia, highlighting the suitability of the device to be employed for this application. Moreover, according to the WHO guidelines for malaria diagnosis, the infection should be confirmed with a parasitological test for which the results must be available within 2 h so that treatment can be provided.^31^ The sensor presented in this study is clearly capable of doing this. By further working on miniaturization (as it has been done with widespread transducer technologies such as electrochemical ones^32^) and taking into consideration that the targeted end user should be instructed on how to employ the sensor, the design of the device could even be simplified for educational purposes^33^ and move forward to application.

The presented results support that the proposed synthetic receptors in combination with the thermal transducer are able to detect the presence of the parasite. An already mentioned advantage is that the device targets the whole infected erythrocyte rather than biomarkers produced from the parasite. Nonetheless, it is equally important to assess the limits of the sensor when it comes to noninfected blood cells in order to avoid false positive results. For this purpose, the functionalized receptors were exposed to infected RBCs (2.7% parasitemia) and noninfected RBCs within the microfluidic device. Figure 2D demonstrates that the sensor provided a specific response toward the infected RBCs and is able to discriminate them from a sample containing healthy RBCs. In previous studies, RBCs imprints were employed to sort different healthy blood types.^34^ The present findings suggest for the first time that IPs have the potential to go further by distinguishing different morphological and chemical features induced in the RBCs under certain anomalies, such as the presence of a parasite, leading to specific binding interactions between iRBCs and the binding pockets in the SIP, which is thermally detected by the sensor. Representative raw data for healthy RBCs in real time can be found on SI, Figure S4.

## Conclusion

This work demonstrates the development of the very first rapid biomimetic test for the direct detection of malaria. By developing imprinted polymers as synthetic receptors for *P. falciparum* and integrating them into a thermal readout platform, a sensor was created that can detect infected RBCs in blood samples. In this way, the study not only provides a proof-of-principle for the creation of such a sensor but also demonstrates its application in biological samples. The sensor meets all the requirements for a PoC test for malaria, the response time of the sensor is about 20 min, and the limit-of-detection of the platform falls within relevant levels for diagnosing low parasitemia concentrations in infected patients. Moreover, data interpretation is straightforward, the benchtop technology requires little instrumentation, which could be easily miniaturized, and the use of artificial receptor layers is known to offer an extended shelf life.

Given the novelty of the application and the findings, this study is presented as a letter. To fully understand the diagnostic potential of the proposed platform, follow-up studies have to be conducted to analyze the sensor’s response to other malaria strains and a preclinical study needs to address its predictive value. The potential impact of these results on the field of biosensing and parasitology is vast and might not only lead to a novel way of diagnosing malaria but, given the versatility of the receptor layers, could also be extended toward the detection of other parasites in future research projects.

## Supporting Information Available

Summary of relevant literature on cell-imprinting, detailed experimental protocols, additional scanning electron images of the imprints, design of the microfluidic device as well as raw data for noncontaminated RBCs measurement (PDF)
