# Supplementary material for: Whole-Cell Thermal Sensor for the Detection of P. falciparum-Infected Erythrocytes: Imprinted Polymers as Synthetic Receptors for the Detection of Malaria
Source: ACS Sens. 2025 Jan 16;10(2):650–6. doi: 10.1021/acssensors.4c02706 (PMC11877496; doi:10.1021/acssensors.4c02706)
Supplement: Supplementary file 1 — se4c02706_si_001.pdf [file se4c02706_si_001.pdf]

Whole-Cell Thermal Sensor for the Detection of *P. falciparum*-infected Erythrocytes: Imprinted Polymers as Synthetic Receptors for the Detection of Malaria.

Rocio Arreguin-Campos *‡[a], Ramayana M. Medeiros Brito‡ †[b], Ana Rafaela Antunes Porto [b], Augusto César Parreiras de Jesus [b, c], Lilian Lacerda Bueno [b, c], Ricardo Toshio Fujiwara [b, c], Hanne Diliën [a], Thomas J. Cleij [a], Kasper Eersels [a], Bart van Grinsven [a]

a Sensor Engineering Department, Faculty of Science and Engineering, Maastricht University, P.O. Box 616, 6200 MD Maastricht, the Netherlands

b Department of Parasitology, Institute of Biological Sciences, Federal University of Minas Gerais, 31270-901, Belo Horizonte, Brazil

c Post-Graduate Program in Health Sciences: Infectious Diseases and Tropical Medicine, Faculty of Medicine, Federal University of Minas Gerais, 30130-100, Belo Horizonte, Brazil

Corresponding author: r.arreguincampos@maastrichtuniversity.nl

Table of Contents

- Summary of relevant studies addressing biomimetic sensors for their application in the health field… ….
…..Table S1

- Detailed materials and methods description… …S2

- Brightfield microscopy images of schizont-infected red blood cells… … … … … Figure S1

- Optical characterization of polymeric imprints on glass… … … … … … … … … … … … Figure S2

- Schematic representation of microfluidic flow cell… … … … … … … … … … … ………. Figure S3

- Representative raw data temperature plot for enrichment experiment of synthetic receptors (SIP) with non-infected red blood cells in real time… … … … … ……..Figure S4

Table S1. Summary of relevant studies addressing biomimetic sensors for their application in the health field.

Analyte	Application	Receptor / Transducer	Sample	Reference
SARS-CoV-2 glycoprotein	COVID-19 biomarker	Polypyrrole / Electrochemical	Buffer	1
Histidine-rich protein	Malaria biomarker	Poly(3,4-ethylenedioxythiphene) / Electrochemical	Buffer	2
Fibrinogen	Platelet aggregation biomarker	Poly (vinylpyridine-co-vinyl imidazole) / Optical	Patient serum samples	3
Prostate-specific antigen	Cancer biomarker	Polyacrylamide / Electrochemical	Buffer	4
Macrophages and cancer cells	Cardiovascular disease and breast cancer biomarkers	Polyurethane / Heat-Transfer Method	Buffer	5
Enterococcus faecium	Infection biomarker	Polypyrrole / Electrochemical	Urine	6
Red blood cells	Blood group sorting	Poly(vinylpyrrolidone) / Micro gravimetric	Blood	7

S2. Detailed materials and methods description

All biological assessment was performed at the Laboratory of Immunobiology and Control of Parasites, Federal University of Minas Gerais (UFMG), Brazil. The blood was collected following the ethical standards of the Research Ethics Committee of the UFMG under the protocol CAAE: #27466214.0.0000.5149.

Fresh blood (O+) was withdrawn from healthy volunteers using EDTA K3 Vacuum Blood Collection System and subsequently submitted to centrifugation at 250 x *g* for 10 min at room temperature. The plasma and the leukocyte ring were removed and three extra washes were performed with 1 mL of 1x PBS in order to completely remove all leukocytes. Subsequently, the erythrocytes pellet was resuspended (1:1 v/v) in Roswell Park Memorial Institute medium (RPMI 1640) with 300 μM hypoxanthine, 11 mM glucose, 40 μg/ mL-1 of gentamicin (Sigma-Aldrich, São Paulo, Brazil) and supplemented with 10% of inactivated human serum type O+ (complete culture medium). The washed blood was kept at 4°C until use.

One mL cryogenic tubes containing *P. falciparum* parasites previously frozen in liquid nitrogen were thawed by adding 1 mL of 3.5% NaCl in RPMI 1640 culture medium without human serum supplementation. The vials were then centrifuged at 250 x g for 8 min at 4°C, the supernatant was discarded and the pellet was transferred to a 15 mL tube and resuspended in 10 mL of complete culture medium. Subsequently, the tube was centrifuged at the same conditions and the supernatant discarded. The pellet of infected erythrocytes was transferred to 25 cm^2 culture flasks containing 4.5 mL of complete culture medium; non-infected RBCs erythrocytes (previously washed blood) were added to complete the final hematocrit to 10% (S1).

The flasks containing the parasites were kept under microaerophilic conditions at 37°C, as previously established[8], with minor modifications[9]. Parasitemia was monitored daily by blood smears stained by the Giemsa dye method and examined under an optical microscope (1000x) to determine the percentage of parasitized red blood cells, using the ratio between the number of infected and non-infected erythrocytes.

Separation of *Plasmodium falciparum* schizont stages. To separate the *P. falciparum* mature schizont stages, a four-step (40, 60, 70, and 80%) Percoll (Sigma-Aldrich, USA) gradient [10]. Briefly, *P. falciparum* cultures with minimum of 10% parasitemia, and at least 80% of schizonts, were transferred to a 15 mL tubes and centrifuged at 250 x g for 5 min at RT, the supernatant was discarded and the pellet resuspended with 1 mL of 10% 10x RPMI 1640 and 5% Sorbitol solution and 100 UI/mL of heparin, the suspension was passed five times through a 23 G 0.6 mm needle, using a 1 mL syringe. The cell suspension was carefully overlaid on top of the Percoll gradient and centrifuged at 10.000 rpm for 30 min at 20°C, without deceleration. The formed ring of parasitized cells containing the schizont stages was collected and transferred to a 15 mL tube and washed in 1x PBS twice, and centrifuged at 3.000 rpm for 5 min. The pellet was then resuspended to 1 mL of 1x PBS, and the number of infected cells counted in the Neubauer chamber. The cell suspension was then fixed by adding 1 mL of 10% buffered paraformaldehyde for 20 min at RT. The infected cells were used to prepare the surface imprinted polymers.

Preparation of synthetic receptors. Graphene oxide powder (Sigma Aldrich) was dispersed into polydimethylsiloxane base resin employing a sonic dismembrator with a probe of 2mm diameter (0.01%). Subsequently, the base containing GO was mixed with the curing agent following the ratio (10:1 (w/w)). The viscous mixture was employed for preparing a stock solution of 10% PDMS in tetrahydrofuran (w/w), which was homogenized by placing it in a vortex mixer until the resin was fully dissolved.

Aluminium chips with a square shape (size of 1cm^2) were spin-coated for 60 s at 5000 rpm with 150 μL of the prepared PDMS-GO stock solutions. In a first step, the thin layers of resin were pre-cured for 10 minutes at 65 °C. Afterwards, the substrates were placed onto a flat surface at room temperature and the parasite suspension (39500 cells in 200 μl 1x PBS buffer) was applied as a droplet on the polymers and left to sediment for 20 min. The substrates with the droplet were then placed together back in the oven 65 °C for 4 hours in order to finalize the curing of the PDMS resin. Once fully cured, the layers were washed with water first to solubilize and rinse the residues of salts on the surface, followed by Ammonium-Chloride-Potassium (ACK) buffer to lyse the red blood cells and make the imprinted cavities free. The same procedure was performed on glass slides that were cut to the same size as the aluminium chips with the aim of employing them for optical characterization.

Optical characterization of polymer imprint's surfaces. Brightfield microscopy was performed on a LEICA DM 750 optical microscope. ImageJ 1.44O (National Institute of Health, Bethesda, MA, USA) was employed to calculate the average surface coverage of cell imprints on the polymeric layers on glass substrates. The surface coverage was determined based on the individual counts of brigthfield images taken on different imprint locations, with an area of 1000 x 800 micrometers per picture.

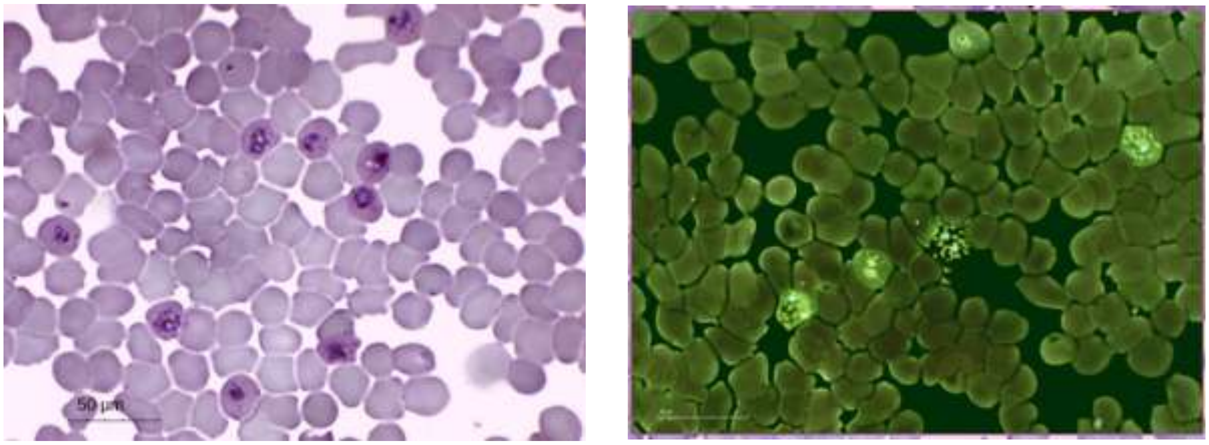

Figure S1. Brightfield microscopy images of schizont-infected red blood cells (stained with Giemsa). These were isolated from younger parasite stages and employed as template for the imprinting process. Scale bars are both 50 µm.

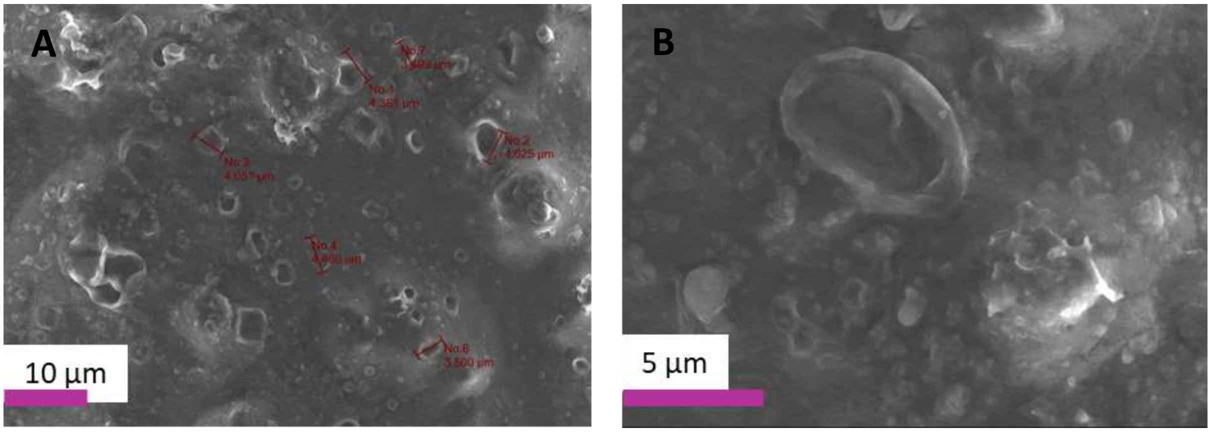

Figure S2. Optical characterization of polymeric imprints on glass. Scanning Electron Microscopy of PDMS-GO imprinted receptors. A) Empty cavities after lysing red blood cells. B) Trapped red blood cell on the polymeric film.

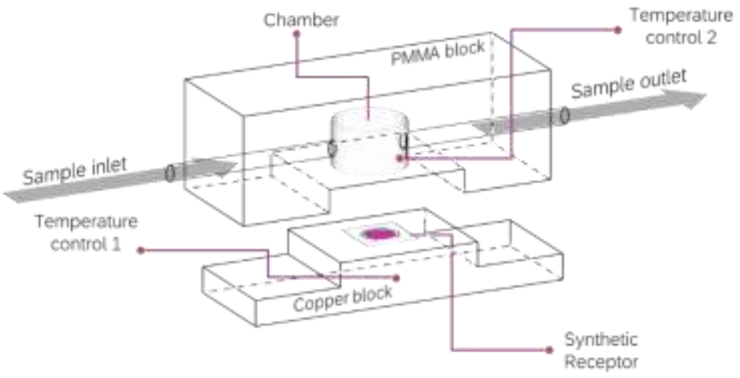

Figure S3. Schematic representation of microfluidic flow cell employed for performing Heat Transfer Method measurements. PMMA: Poly (methyl methacrylate).

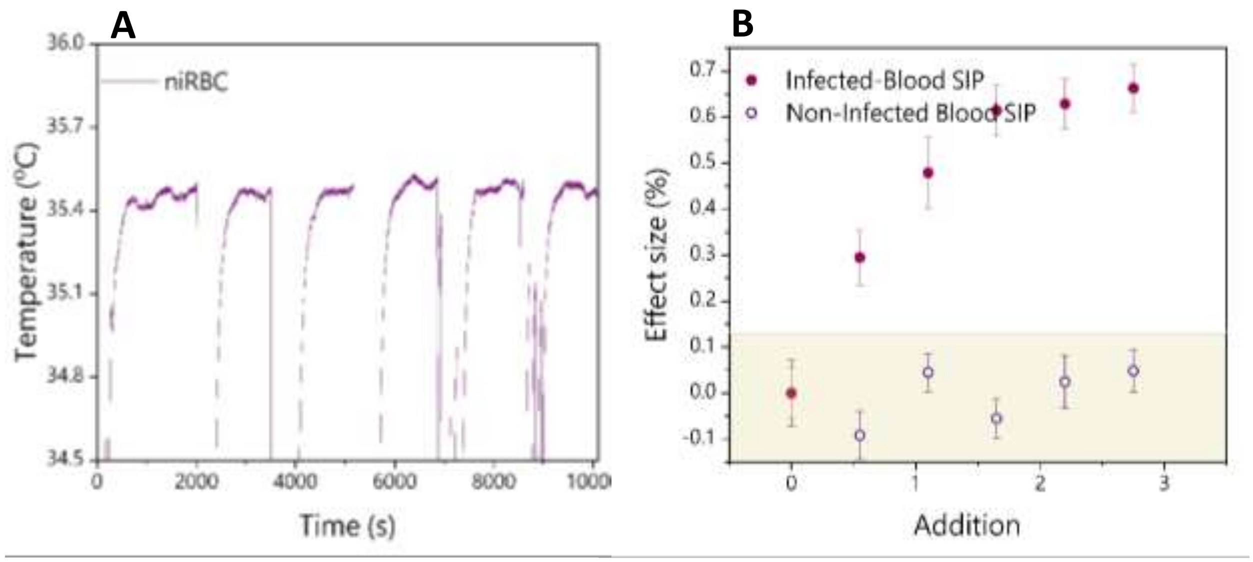

Figure S4. A) Representative raw data temperature plot for enrichment experiment of synthetic receptors (SIP) with non-infected red blood cells in real time. B) Comparison dose-response curve against infected red blood cells. Each addition represents 0.5% parasitemia for the infected enrichment experiment.

References

(1) Ratautaite, V.; Boguzaite, R.; Brazys, E.; Plausinaitis, D.; Ramanavicius, S.; Samukaite-Bubniene, U.; Bechelany, M.; Ramanavicius, A. Evaluation of the Interaction between SARS-CoV-2 Spike Glycoproteins and the Molecularly Imprinted Polypyrrole. Talanta 2023, 253, 123981. https://doi.org/10.1016/j.talanta.2022.123981.

(2) Glória, J. C.; Oliveira, D. S.; Gandarilla, A. D.; Barcelay, Y. R.; Mariúba, L. A. M.; Nogueira, P. A.; Brito, W. R.; Moreira, F. T. C. Liquid Redox Probe-Free Plastic Antibody Development for Malaria Biomarker Recognition. ACS Omega 2024. https://doi.org/10.1021/acsomega.4c04543.

(3) Çimen, D.; Üzek, R.; Günaydın, S.; Denizli, A. Real-Time Detection of Fibrinogen via Imprinted Recognition Sites. ChemistrySelect 2021, 6 (35), 9435–9441. https://doi.org/10.1002/slct.202101942.

(4) Karami, P.; Bagheri, H.; Johari-Ahar, M.; Khoshsafar, H.; Arduini, F.; Afkhami, A. Dual-Modality Impedimetric Immunosensor for Early Detection of Prostate-Specific Antigen and Myoglobin Markers Based on Antibody-Molecularly Imprinted Polymer. Talanta 2019, 202, 111–122. https://doi.org/10.1016/j.talanta.2019.04.061.

(5) Eersels, K.; Van Grinsven, B.; Ethirajan, A.; Timmermans, S.; Jiménez Monroy, K. L.; Bogie, J. F. J.; Punniyakoti, S.; Vandenryt, T.; Hendriks, J. J. A.; Cleij, T. J.; Daemen, M. J. A. P.; Somers, V.; De Ceuninck, W.; Wagner, P. Selective Identification of Macrophages and Cancer Cells Based on Thermal Transport through Surface-Imprinted Polymer Layers. ACS Appl. Mater. Interfaces 2013, 5 (15), 7258–7267. https://doi.org/10.1021/am401605d.

(6) Oğuzhan Kaya, H.; Tekintaş, Y.; Kurul, F.; Cetin, A. E.; Nur Topkaya, S. Targeted Microorganism Detection with Molecularly Imprinted Polymer Biosensors. J. Electroanal. Chem. 2024, 971, 118575. https://doi.org/10.1016/j.jelechem.2024.118575.

(7) Seifner, A.; Lieberzeit, P.; Jungbauer, C.; Dickert, F. L. Synthetic Receptors for Selectively Detecting Erythrocyte ABO Subgroups. Anal. Chim. Acta 2009, 651 (2), 215–219. https://doi.org/10.1016/j.aca.2009.08.021.

(8) Trager, W.; Jensen, J. B. Human Malaria Parasites in Continuous Culture. Science (80-.). 1976, 193 (4254), 673–675. https://doi.org/10.1126/science.781840.

(9) Andrade-Neto, V. F.; Brandão, M. G. L.; Stehmann, J. R.; Oliveira, L. A.; Krettli, A. U. Antimalarial Activity of Cinchona-like Plants Used to Treat Fever and Malaria in Brazil. J. Ethnopharmacol. 2003, 87 (2–3), 253–256. https://doi.org/10.1016/s0378-8741(03)00141-7.

(10) Fernandez, V.; Treutiger, C. J.; Nash, G. B.; Wahlgren, M. Multiple Adhesive Phenotypes Linked to Rosetting Binding of Erythrocytes in Plasmodium Falciparum Malaria. Infect. Immun. 1998, 66 (6), 2969–2975. https://doi.org/10.1128/IAI.66.6.2969-2975.1998.
